# Telemedicine framework to mitigate the impact of the COVID-19 pandemic

**DOI:** 10.1016/j.jtumed.2020.12.010

**Published:** 2021-01-14

**Authors:** Gopi Battineni, Graziano Pallotta, Giulio Nittari, Francesco Amenta

**Affiliations:** Telemedicine and Telepharmacy Centre, School of Medicinal and Health Products Sciences, University of Camerino, Camerino, Italy

Dear Editor,

With more than 92 million infected cases, including about 2 million deaths, globally the novel coronavirus (COVID-19)^1^ pandemic has shattered the health systems of most nations worldwide. Moreover, many of these systems are unprepared due to a lack of infrastructure and methods in place to guarantee complete health coverage. In view of the high pressure on health systems by the pandemic, reliable medical care is needed. This can be obtained via comprehensive digital techniques, especially in treatment and decision making during a severe pandemic such as COVID-19. The COVID-19 pandemic has triggered all healthcare authorities in one direction to promote the use of digital technologies. Despite some legal and ethical issues, most medical experts argue that telemedicine is a potential source of health care during global epidemics. The implementation of telemedicine systems with the objective of monitoring mild or asymptomatic cases would reduce the high volume of incoming patients. It could limit the cluster of cases and avoid the risk of infection for both patients and health professionals.

Telemedicine and telemonitoring solutions can treat patients in an emergency without physical presence at a hospital. With video conferencing, telemedicine systems enable direct communication with patients by smartphone or PC. The entire system should be managed by specially designed software to guarantee stable connections, information flow, and data security.^2^ These systems are a mixture of medical devices, which will automatically record symptomatic information and assess the severity of a medical problem. Numerous efforts are required by all countries to ensure that epidemics such as the COVID-19 pandemic can be managed effectively. From this perspective, if telemedicine is fully integrated into a national health system, it could prove a valid instrument on its own to guarantee continuity of care and reduce the risk of contagion, especially among health professionals. It would also translate into reduced overcrowding of health facilities and result in only a minor depletion of doctors and nurses. However, we also observed unintended problems for doctors, patients, and society at large. In particular, in the U.S. population during COVID-19, disparities for telemedicine access were found in ethnic and racial minorities, the elderly, and those on a low income, among others.

Western healthcare systems are based on the model of in-person interactions between patients and their clinicians.^3^ This method of assistance contributes to the spread of virus not only to patients but also to health professionals who are carrying out the patient monitoring. A digitised care strategy should be applied in situations of isolation, lockdown, and limitation of movement, especially in those areas where healthcare facilities are not in close vicinity, or for those patients whose mobility is reduced. The validity and reliability of telemedicine systems have been confirmed by the example of some remote areas of China.^4^ For instance, infected patients in home isolation are able to record body temperatures or other metrics with simple devices like a thermometer through video conference with their personal doctor to avoid the risk of either being infected.^4^^,^^5^

Telemedicine has already been proven as an effective solution in past pandemics, such as the Severe Acute Respiratory Syndrome Coronavirus, Middle East Respiratory Syndrome Coronavirus, Ebola, and Zika. For instance, if a person is exposed to mild COVID-19 symptoms, doctors recommend a home quarantine. However, a greater risk of psychological issues like anxiety, depression, and distress is possible. To reduce this risk, different computer mechanisms and digital technologies are recommended. Yet, to date, there is no reliable and authenticated telemedicine system to mitigate the risk of infection. In this work, we present a conceptual framework (Figure 1) for the remote monitoring of infected and suspected COVID-19 patients. This integrated system may guarantee controlled management not only of positive patients but also asymptomatic and chronically ill patients in the COVID-19 outbreak and/or similar emergencies.

**Figure 1:**
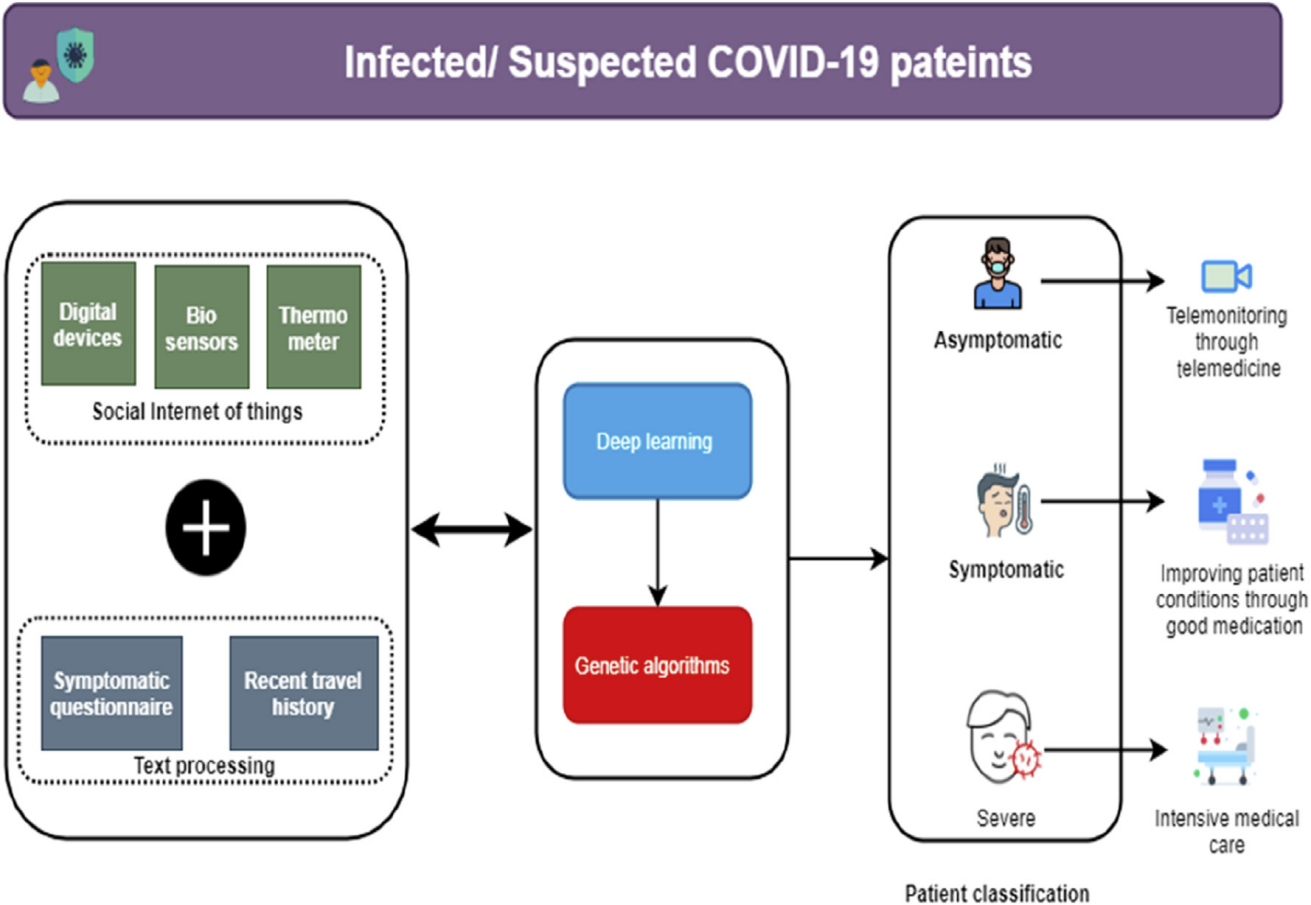
Telemedicine monitoring framework to assist the COVID-19 infected/suspected population.

The proposed model is framed with both Social Internet of things (SIoT) and Artificial Intelligence (AI) techniques such as text processing, deep learning, and genetic algorithms. The SIoT is used to collect the symptomatic information of COVID-19 infected patients. Digital devices, biosensors, and thermometers can help to provide daily information such as heart rate, blood pressure, body temperature, galvanic skin response. After having collected the required information, with a mixture of deep learning, text processing, and genetic algorithms the model framework is employed to create a comprehensive classification model to verify patient condition (symptomatic, asymptomatic, or severe conditions). Three types of telemedicine services can be offered within the proposed modelling framework as mentioned below.

- If the patient is asymptomatic, telemedicine monitoring will be continued until the incubation period is complete.
- If the patient is symptomatic but mild to moderate, continuous expert contact will be maintained through video conferencing to mitigate further symptoms.
- If the patient has severe conditions, immediate information can be conveyed to emergency medical services for the ultimate provision of intensive care.^6^

## Source of funding

This work was supported by a grant No. 1508/2020 from the ITF Trust, London (UK).

## Conflict of interest

The authors have no conflict of interest to declare

## Ethical approval

The authors did not have permission for direct communication with human participants in the study and no ethical issues were encountered during the study presentation.

## Authors' contributions

GP and GB conceived and designed the study, conducted research, provided research materials, and collected and organised data; GN analysed and designed the framework; GP, GB, and FA wrote the initial and final drafts of the article and provided logistic support. All authors have critically reviewed and approved the final draft and are responsible for the content and similarity index of the manuscript.
